# Pentalogy of Cantrell: a case report

**DOI:** 10.1259/bjrcr.20140002

**Published:** 2015-06-29

**Authors:** A R Patil, L S Praveen, V Ambica

**Affiliations:** ^1^Department of Radiodiagnosis, Apollo Hospitals, Bangalore, India; ^2^Department of Radiodiagnosis, Clumax Diagnostics, Bangalore, India; ^3^Department of Obstetrics and Gynaecology, Clumax Diagnostics, Bangalore, India

## Abstract

Pentalogy of Cantrell is a rare condition comprising anterior diaphragmatic defect, ventral abdominal wall defect, pericardial defect, intracardiac anomalies and lower sternal defect. Both sporadic and genetic causes are proposed. Prognosis depends on the severity of the defects and the associated cardiac anomalies. Two-dimensional sonography is sufficient for the diagnosis of this condition.

## Clinical presentation

A 24-year-old primigravida had come for her routine anomaly scan at 24 weeks of gestational age. The early obstetric scan had not been performed. Her prenatal or antenatal course was unremarkable. No history of febrile illness or intake of any medications was elicited.

## Investigations/Imaging findings

On sonographic examination, the foetus showed symmetrical intrauterine growth restriction with the biparietal diameter and femur length corresponding to 22 weeks. The intracranium and long bones were anatomically normal with no obvious anomalies.

The foetus had a large supraumbilical anterior abdominal wall defect with herniation of liver, stomach and small intestines (see Figures 2b and 4a). The herniated organs were contained by a thin membrane, which was hardly perceptible on ultrasonography.

**Figure 1.**
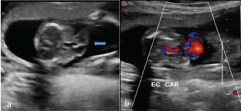
(a) Axial section of the foetal chest shows ectopia cordis (arrow) with absent pericardium. (b) Same section with colour Doppler.

**Figure 2.**
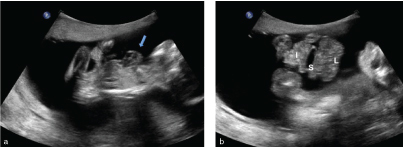
(a) Sagittal scan of the foetus shows ectopia cordis with the apex of the heart (arrow) pointing towards the chin. (b) Parasagittal section shows herniation of liver (L), stomach (S) and intestines (I) floating in the amniotic fluid.

**Figure 3.**
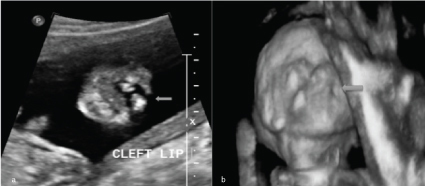
(a) Two-dimensional sonography depicting left paramedian cleft lip (arrow). (b) Cleft lip on three-dimensional sonography (arrow).

There was ectopia of the foetal heart (ectopia cordis) with absent pericardium (Figure 1a,b). The apex of the ectopic heart was pointing towards the chin of the foetus (Figure 2a). The cardiac activity was, however, normal, ranging 150–155 beats per minute. The cardia had a large ventricular septal defect. The foetal kidneys were normal. The spine had reversed curvature owing to the large omphalocele. Additionally, there were left paramedian cleft lip and palate (Figure 3a,b). Liquor volume was normal.

Based on this conglomeration of findings, a final diagnosis of “pentalogy of Cantrell” was arrived at.

Pentalogy of Cantrell is a rare condition with a reported incidence of <1 in 100,000 and a 2:1 male predominance.^1^ The five components of the pentalogy are anterior diaphragmatic defect, ventral abdominal wall defect, pericardial defect, intracardiac anomalies and lower sternal defect.^2^ Toyama^1^ proposed the following criteria in diagnosing the condition:

1. certain if all five defects are present
2. probable if four are present (including intracardiac and ventral abdominal wall)
3. incomplete if variable combinations are seen (always including sternal abnormalities).

Our case was the complete/severe type of pentalogy of Cantrell with all five components present with truncus arteriosus and ventricular septal defect as the cardiac abnormalities.

The occurrence of pentalogy is believed to be sporadic. Gene expression studies in mouse and human embryonic tissues have led to consideration of genetics in the disease pathogenesis. Some investigators have proposed *BMP2 *(bone morphogenetic protein 2) gene mutations as a likely cause for this condition since these genes are responsible for the normal development of midline structures.^3^

A case report by Steiner et al^4^ suggested the role of *ALDH1A2* in the pathogenesis of pentalogy. *ALDH1A2,* located in chromosome 15, which encodes the enzyme retinaldehyde dehydrogenase type 2, is critical for the conversion of vitamin A into all trans-retinoic acid. Retinoic acid is known to be a powerful morphogen, important during early development for axial patterning and in later development for organogenesis, and has an established role in the pleuroperitoneal folding as in diaphragm embryogenesis.

Cantrell et al^2^ had proposed that this entity results from failure of development of a lateral mesodermal segment at 14–18 days of embryonic life, which, in turn, would cause failure of development of the transverse septum of the diaphragm and a failure in the ventromedial migration of the paired mesodermal folds of the upper abdomen, leading to midline fusion defects manifesting in varying severity. Additionally, the presence of cleft lip, cleft palate and craniorachischisis could represent arrest in embryonic midline.

The associated anomalies that have been reported with pentalogy of Cantrell include craniofacial and central nervous system anomalies such as cleft lip and/or cleft palate, encephalocele, hydrocephalus and craniorachischisis.^5^ Thoracoabdominal organ abnormalities include lung hypoplasia, adrenal hypoplasia, gallbladder agenesis, single renal agenesis, polysplenia, malrotation of the colon, herniation of bowel into pericardium, bladder exstrophy, undescended testes and bilateral inguinal hernia, and limb defects include club foot, absence of tibia, radius and hypodactyly.

The cardiac abnormalities in pentalogy include ventricular septal defects, Ebstein’s anomaly, truncus arteriosus, transposition of great vessels, single atrium and atrioventricular canal.

Two-dimensional sonography is sufficient in diagnosing this condition. Three-dimensional sonography might help in better depiction of the extent of the abnormalities to the clinician and the patient, and, hence, decide the prognosis and feasible treatment method.^6^

The treatment strategy depends on the size, the content and the state of the omphalocele defect and the associated heart anomalies. In the less severe variety of pentalogy of Cantrell, abdominal wall and diaphragmatic defect reconstruction can be performed followed by chest wall reconstruction and corrective cardiac surgery.

In the severe form of pentalogy of Cantrell with ectopia cordis, the surgical correction is often difficult secondary to hypoplasia of the thoracic cage and inability to enclose the ectopic heart.

## Differential Diagnosis

The main differential diagnosis is the omphalocele–exstrophy–Jimperforate anus–spinal defects complex, which is characterized by a combination of omphalocele, exstrophy of the bladder, an imperforate anus and spinal defects. Isolated thoracic cardiac ectopy, ectopia cordis associated with amniotic band syndrome, body stalk abnormality and isolated omphalocele are other differentials to be considered.

## Treatment

Considering the poor prognosis and complexity of anomalies along with hypoplastic lungs (which may pose a threat even if treatment of ectopia cordis is considered), termination was advised in our case.

Medical termination of pregnancy was performed by induction.

## Outcome and follow-up

**Figure 4.**
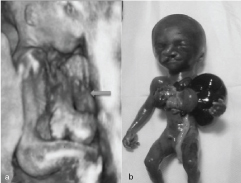
(a) Three-dimensional sonography of the foetus depicts herniation of abdominal viscera (arrow) and cardia. (b) Abortus showing ruptured omphalocele, ectopia cordis, cleft lip and low-set ears.

The foetus was male with a large ruptured supraumbilical omphalocele with herniation of liver, gallbladder, stomach and small intestines (Figure 4b). The spleen was intra-abdominal. Both the kidneys were normal. There was bifid sternum with ectopia cordis and absent pericardium. The diaphragm had a defect anteriorly. There was a large ventricular septal defect with aorta and pulmonary artery originating from a common trunk. The lungs were hypoplastic. Low-set ears and left paramedian cleft lip and palate were present. The spine was intact. The intracranium revealed no obvious abnormalities.

Further counselling regarding the next pregnancy planning and prenatal follow-up was provided to the couple.

## Learning Points

The five components of the pentalogy are anterior diaphragmatic defect, ventral abdominal wall defect, pericardial defect, intracardiac anomalies and lower sternal defect.

Two-dimensional ultrasound is the best modality to diagnose and decide the prognosis of the condition. Awareness of this rare condition enables proper counselling to the parents.
